# The Next Frontier in Tuberculosis Investigation: Automated Whole Genome Sequencing for *Mycobacterium tuberculosis* Analysis

**DOI:** 10.3390/ijms25147909

**Published:** 2024-07-19

**Authors:** Justin H. J. Ng, Lina Castro, Andrew Gorzalski, Adam Allred, Danielle Siao, Edwina Wong, Andrew Lin, Shadi Shokralla, Mark Pandori, Godfred Masinde, Ramin Khaksar

**Affiliations:** 1 Clear Labs, Inc., 1559 Industrial Road, San Carlos, CA 94070, USA; justin.ng@clearlabs.com (J.H.J.N.);; 2 San Francisco Public Health Laboratories, 101 Grove Street, Room 419, San Francisco, CA 94102, USA; 3 Nevada State Public Health Laboratory, 1664 North Virginia Street, Reno, NV 89557, USA

**Keywords:** *Mycobacterium tuberculosis*, whole genome sequencing, automation, antimicrobial resistance, decentralized diagnostics

## Abstract

A fully automated bacteria whole genome sequencing (WGS) assay was evaluated to characterize *Mycobacterium tuberculosis* (MTB) and non-tuberculosis *Mycobacterium* (NTM) clinical isolates. The results generated were highly reproducible, with 100% concordance in species and sub-lineage classification and 92% concordance between antimicrobial resistance (AMR) genotypic and phenotypic profiles. Using extracted deoxyribonucleic acid (DNA) from MTB clinical isolates as starting material, these findings demonstrate that a fully automated WGS assay, with a short turnaround time of 24.5 hours, provides timely and valuable insights into MTB outbreak investigation while providing reliable genotypic AMR profiling consistent with traditional antimicrobial susceptibility tests (AST). This study establishes a favorable proposition for the adoption of end-to-end fully automated WGS solutions for decentralized MTB diagnostics, thereby aiding in World Health Organization’s (WHO) vision of tuberculosis eradication.

## 1. Introduction

*Mycobacterium tuberculosis* (MTB) is a preventable and usually curable disease, and yet it causes tens of millions of new cases of tuberculosis (TB), resulting in more than one (1) million deaths every year worldwide [1]. Multidrug-resistant (MDR) tuberculosis is a major health problem and seriously threatens worldwide TB control and prevention initiatives. Since 2020, there have been an estimated 465,000 Rifampicin-resistant (RR) tuberculosis cases, with 78% of these cases classified as multidrug-resistant (MDR). This indicates that the MTB strains causing these cases are resistant to both Rifampicin and Isoniazid [2]. There are two main challenges with MDR-MTB: underdiagnosis of clinical cases and poor treatment outcomes, both of which lead to more serious clinical complications. Collectively, the failure to identify and successfully treat these cases leads to further exacerbation and transmission of MDR-MTB strains.

The WHO has emphasized that accurate diagnosis and characterization of MTB is the key strategy for successful treatment and eradication of complicated TB cases [3]. Rapid genotypic-based diagnostic tools, such as GeneXpert, have been widely adopted as they are faster and cheaper than traditional culture-based diagnostic susceptibility testing. However, outbreaks caused by MDR strains with novel antimicrobial resistance (AMR) profiles may not be picked up by these assays, which focus on a limited number of targets. This underscores the importance of employing assays with a genome-wide approach that covers a broader range of resistance determinants [4]. Although molecularly targeted assays have shown great value in MTB AMR characterization [2], these methods are still dependent on known genotypic and mutation profiles [1]. Failure to detect novel AMR genotypes and mutations within the MTB genome can lead to inaccurate prescription of patient treatment regimes, posing a risk of treatment failure [3]. Whole genome sequencing (WGS) is an agnostic approach to screening the entire pathogen genome for the detection of specific single nucleotide polymorphisms (SNPs) to discriminate known MTB complex (MTBC) (sub)lineages. This approach not only enables the presence/absence of AMR genes but also the agnostic screening and characterization of AMR loci. WGS has significant potential to replace traditional culture-based phenotypic diagnostic tests, which can typically take weeks to months [5]. By reducing turnaround time to less than two (2) weeks, it will improve antibiotic stewardship for first-line drugs in TB treatment.

Widespread adoption and implementation of this methodology remain challenging due to a lack of necessary infrastructure, the high cost of entry, and the requirement for technical expertise to process the samples and analyze the generated sequencing data [4]. Integrating next-generation (NGS) technologies with automation will alleviate these challenges and has been proven successful for application in the food safety and infectious diseases space [6,7,8,9]. Automation imparts multiple benefits to existing manual workflows. It can improve results consistency and reproducibility by reducing the number of human touch points and eliminating human/random errors in the process. Researcher efficiency can also be improved by eliminating the need for manual and routine tasks. Automation enables the performance of workflows around the clock without human supervision or intervention, further reducing turnaround time from sample input to result output. Finally, it aids in improving workplace safety by eliminating the exposure of laboratorians to potentially harmful chemicals [10]. In this study, we evaluated the accuracy and specificity of a fully automated bacteria WGS assay for the characterization of MDR-MTB isolates from reference collections as well as clinical isolates.

## 2. Results

### 2.1. WGS Characterization of Mycobacterium tuberculosis (MTB) and Non-Tuberculosis Mycobacterium (NTM) Isolates

A total of 86 samples (44 unique isolates) were sequenced in this study using the fully automated bacteria WGS assay. MTBs and NTMs isolates were assembled, with their taxons correctly identified down to the species level and their sub-lineages characterized (Table 1). AMR genotypic profiles and associated drug resistance predictions were determined and summarized in Table 2.

### 2.2. Robustness and Reproducibility of the Fully Automated Bacteria WGS Assay

Three (3) strains of clinical MTB isolates provided by NSPHL, together with four (4) strains each of MTB and NTM isolates procured from reference repositories (ATCC and BEI Resources), were used to assess the reproducibility and robustness of the fully automated bacteria WGS assay. Replicates were sequenced across three (3) different systems, and the results are summarized in Table 3. Lyve-SET analysis of NSPHL Strains 8, 12, and 17 reported no SNP distance between replicates (see Table S1).

### 2.3. Study Site-Specific Clinical Isolate Relatedness Analysis

**Figure 1:**
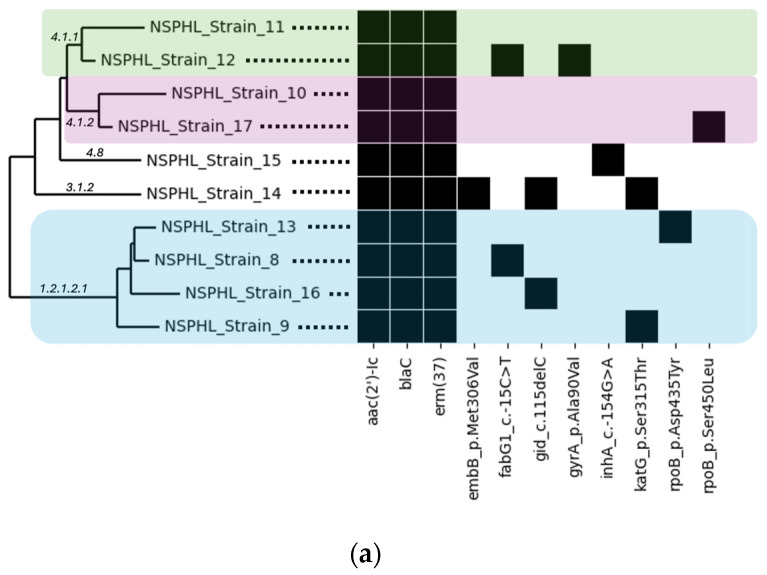

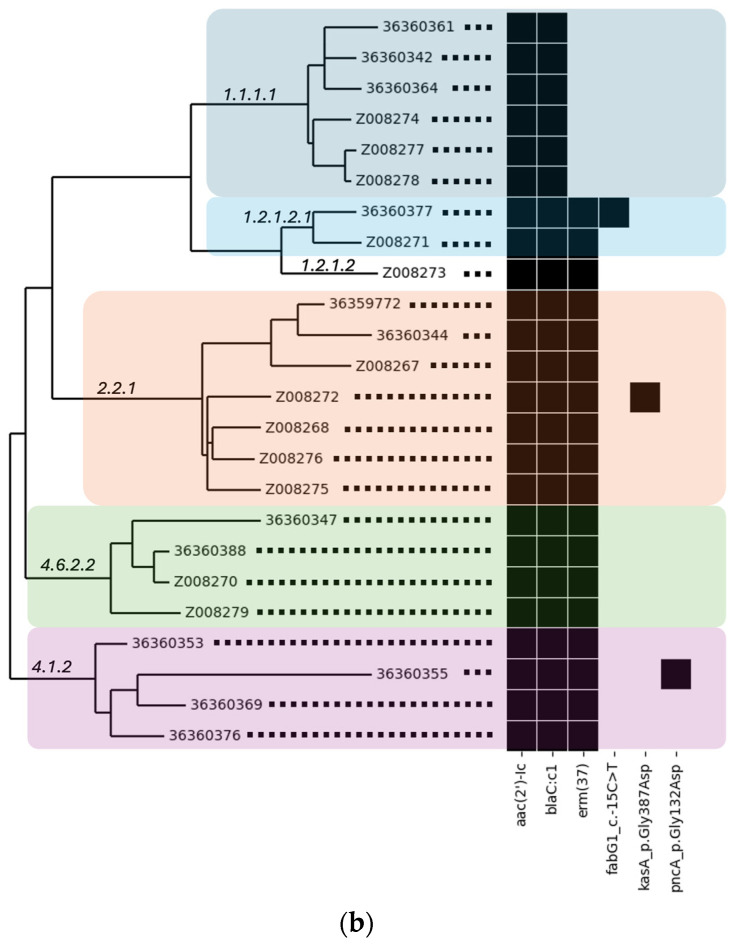
Phylogeny of Clinical Isolates from (**a**) Nevada State Public Health Laboratory and (**b**) San Francisco Public Health Laboratory with Corresponding AMR Genotypic Profile.

Whole genome phylogenetic analysis of clinical isolates was performed to predict MTB clusters from NSPHL and SFPHL (Figure 1a and Figure 1b, respectively). Phylogenetic groupings for the genomes within each study site were consistent with pairwise SNP distances as computed by Lyve-SET for the same genomes (Tables S1 and S2). Isolates within the same cluster shared the same node and showed lower SNP distances, while isolates from different clusters had higher SNP distances. For example, NSPHL Strains 10 and 17, which were within a single cluster, had an SNP distance of 229, while NSPHL Strains 10 and 13, which were between clusters, had an SNP distance of 1979 (see Table S1). SFPHL clinical isolates 36359772, 36360344, and Z008267 were within the same cluster and had zero (0) SNP distance, while isolates Z008272 and Z008273 were from different clusters and had an SNP distance of 2013 (see Table S2).

### 2.4. Comparison of Genotypic and Phenotypic AMR Results

Genotypic AMR profiles from the WGS data of the clinical isolates were compared with phenotypic data from AST provided by the study sites. The predicted AMR drug classes, based on the AMR gene profiles, perfectly matched the TB-specific AST phenotypic results from NSPHL clinical isolates (Table 4). The vast majority of clinical MTB isolates from SFPHL were sensitive to Streptomycin, Isoniazid, Rifampicin, Ethambutol, and Pyrazinamide, aligning perfectly with the genotypic AMR profiles. Three (3) isolates exhibited non-concordance between their genotypic AMR profiles and AST results. Isoniazid resistance was predicted for 36360377, while Pyrazinamide resistance was predicted for 36360355 and Z008272. AST results indicated that all three (3) isolates were sensitive to all TB first-line drugs (see Table S3).

## 3. Discussion

Although phenotypic confirmation of TB is still the gold standard and necessary for the testing of resistance to anti-TB drugs, genetic sequencing at reference-level laboratories is slowly gaining popularity [11]. WGS is, without a doubt, a more powerful tool and can play a major role in the diagnosis of MTB drug resistance when compared with PCR-based methods. It has the advantage of enabling the identification of “off-targets” or new candidate resistance mutations when facing AST discrepancies or resistance to new and/or repurposed drugs, for which resistance catalogs are still being developed [12]. In recent years, the application of WGS for TB diagnosis has rapidly progressed from an academic research-only perspective to routine patient care in clinics, population-level surveillance, and the formulation of public health intervention strategies [13,14,15,16]. These strategies, in turn, help with more effective antibiotic stewardship as well as the implementation of pathogen-based precision medicine treatments for TB [17].

Integrating WGS with automation will help reduce the complexity and hands-on time required for the experimental workflow, further reducing turnaround time for diagnosis and increasing actionability. This would open up the potential of providing a turnkey solution for widespread adoption as well as democratizing WGS technology for TB diagnosis in laboratories, hospitals, and clinics with limited personnel and technical expertise. Here, we showed that a fully automated bacteria WGS assay, using extracted DNA from isolates as starting material, is capable of generating highly robust and reproducible results with a turnaround time of approximately 24.5 h as compared to the 6–10 days needed for manual workflow. The fully automated assay was able to achieve a short turnaround time by enabling around-the-clock operation without the need for human supervision and intervention, which is not the case for manual workflows restricted by stipulated laboratorian operating hours. Sample registrations, and the loading of samples and consumables took approximately 30 min in total. Data from sequencing the entire MTB genome provided high-resolution information, conferring unique insights into AMR genotypic profiles of individual isolates, as well as how they were related to one another.

A greater depth of information helped tease out the intricate relationships of isolates classified within the same sub-lineage. For example, in the case of SFPHL clinical isolates, although isolates 36360342, 36360361, 36360364, Z008274, Z008277, and Z008278 are all from sub-lineage 1.1.1.1, isolates 36360342, 36360361, and 36360364 sub-clustered together and had a SNP distance of zero (0) (see Figure 1b and Table S2). Therefore, we hypothesized that these three (3) isolates might be the same strain or from the same origin. Isolates 36360347, 36360388, and Z008270 had an SNP distance of five (5) when compared to one another, while isolate Z008279 had an SNP distance of between 173 and 194 when compared against these three (3) isolates (see Figure 1b and Table S2). We, therefore, hypothesized that isolates 36360347, 36360388, and Z008270 might be derived from the same outbreak while isolate Z008279 is from a separate event, even though they share the same AMR profile and 4.6.2.2 sub-lineage classification.

Correlations between genotypic and phenotypic profiles of AMR have been widely debated, with varying concordances reported dependent on drug classes and organisms tested [18,19,20,21]. Similarly, high but imperfect concordance has been reported for MTB [12,22]. The findings in this study are consistent with those in the literature. Clinical isolates from NSPHL revealed 100% concordance between WGS genotypic AMR profiles and AST phenotypes, further supporting the use case of applying WGS for comprehensive TB diagnosis in clinical settings. Out of the 24 clinical isolates from SFPHL, 21 showed agreement between genotypic and phenotypic AMR profiles, while three (3) isolates had some discrepancies. These three (3) isolates had AMR genotypes predicting resistance against Isoniazid or Pyrazinamide, despite AST data indicating susceptibility to both drugs in all three cases. The authors emphasize that achieving consistent data without human error is a significant achievement, far outweighing the few discrepancies. This outcome strongly endorses the use of a fully automated WGS assay in TB diagnosis. While the results from the genotypic AMR profile may impact treatment options, in this case, these results would encourage clinicians to proceed with caution in treatment selection. This cautious approach further promotes antibiotic stewardship efforts by choosing and tailoring appropriate first-line drug treatments for specific TB cases.

## 4. Materials and Methods

### 4.1. Sample Source

Bacterial isolates or extracted DNA from reference MTB and non-tuberculosis *Mycobacterium* (NTM) were procured from ATCC or BEI Resources, NIAID, and NIH. DNA extracted from de-identified clinical isolates was obtained from two (2) study sites: the Nevada State Public Health Laboratory (NSPHL) and the San Francisco Public Health Laboratory (SFPHL). Details of the isolates and strains used in this study are summarized in Table 5.

### 4.2. Isolate Cultures and DNA Extraction

Non-tuberculosis *Mycobacterium* (NTM) and an attenuated MTB strain (NR-122) extracted at Clear Labs, Inc. were first grown on Löwenstein–Jensen (LJ) agar slants (Hardy Diagnostics, Santa Maria, CA, USA) incubated at 35 °C ± 2 °C for 7–10 days. Bacterial isolates were resuspended in Clear Labs’ Resuspension Buffer v2.0 (Clear Labs, Inc., San Carlos, CA, USA) and heat-inactivated at 95 °C for 30 min. MTB cell suspensions were lysed and DNA extracted using a proprietary Clear Labs in-house-developed extraction protocol. Extracted DNA was stored at −80 °C until use.

MTB clinical samples provided by NSPHL were grown on LJ agar slants, resuspended in HPLC water, and inactivated by boiling for 30 min. DNA was then extracted from heat-inactivated cultures using the Maxwell RSC Cultured Cell Kit (Promega, Madison, WI, USA) or DNeasy Blood & Tissue QIAcube Kit (QIAGEN, Venlo, The Netherlands). Extracted DNA was stored at −80 °C until use.

MTB clinical samples provided by SFPHL were grown on LJ agar slants, resuspended in HPLC water, and inactivated by boiling for one (1) hour. DNA extractions were then performed using the MagNA Pure 24 Total NA Isolation Kit (Roche Life Sciences, Indianapolis, IN, USA) or the EZ1&2 Virus Mini Kit v2.0 (Qiagen, Germantown, MD, USA), as detailed in Table 1. Extracted DNA was stored at −80 °C until use.

### 4.3. DNA Quantification

Extracted DNA was quantified on a Qubit™ 4 Fluorometer using the Qubit 1x dsDNA High Sensitivity Kit according to the manufacturer’s instructions (Thermo Fisher Scientific, Waltham, MA, USA).

### 4.4. Workflow of a Fully Automated Platform for WGS of Extracted DNA from MTB and NTM

**Figure 2:**
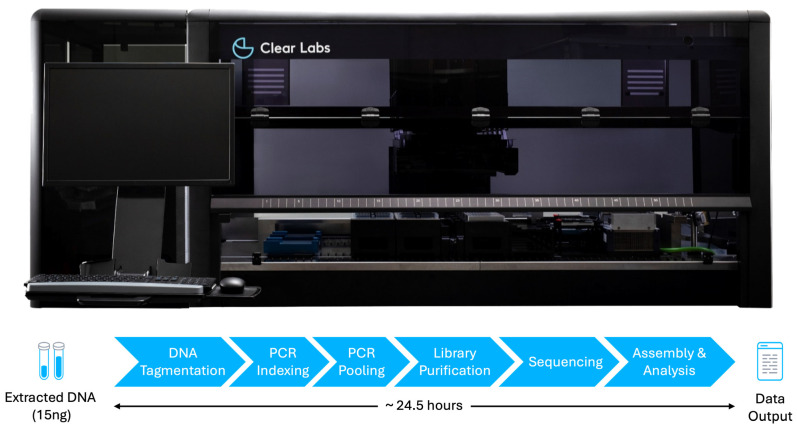
Schematic representation of the fully automated bacterial WGS workflow using extracted DNA as sample input.

The extracted DNA from all isolates was first diluted to a concentration of 0.5 ng/µL in a 30 µL volume (total DNA input yield of 15 ng). Samples were prepared, sequenced, and analyzed using the “Low DNA Input” protocol of the Clear Dx™ Microbial Surveillance WGS v2.0 application on the fully automated and integrated Clear Dx™ platform, which comprised liquid handling robotics, thermal cyclers, and sequencers (Figure 2). The Clear Dx™ Microbial Surveillance WGS assay is for research use only and provides additional information that can potentially infer clinical decisions but does not recommend clinical decisions as result output. Briefly, samples, reagents, and consumables were first loaded onto the instrument according to the manufacturer’s instructions (Clear Labs, San Carlos, CA, USA). Up to 12 samples per run were then registered using the ClearView App software, version 4.6.2 with “*Mycobacterium* (4.4 MB)” selected as “Organism” and “30–40×” selected as “Coverage.” Upon pushing the “Start Run” button, the fully automated system performs library preparation using the 2 × 150 bp chemistry, followed by the loading of sequencing cartridges onto the Illumina iSeq100 sequencers (Illumina, Inc., San Diego, CA, USA) as a part of the end-to-end workflow. After the completion of sequencing, raw sequencing data were automatically uploaded to the cloud for bioinformatics analysis. The final output generated by the workflow comprised compressed FASTQ files, sample identity (genus and/or species level), and quality metrics for each isolate. The fully automated end-to-end workflow took approximately 24.5 h in total.

### 4.5. Data Analysis

The TheiaProk version 1.3.0 bioinformatics pipeline was used for bioinformatics analysis [23] once sequencing reads were generated from the fully automated end-to-end workflow. Briefly, paired-end Illumina reads in FASTQ format generated by the fully automated bacteria WGS assay were first trimmed and quality filtered using Trimmomatic and fastq_scan pipelines. Cleaned reads were then de novo assembled using the Shovill algorithm, assembly quality control (QC) was checked by QUAST version 5.2.0 and BUSCO version 5.7.1 pipelines, and the taxonomic assignment was performed using GAMBIT version 1.0.1. Further downstream analysis includes genome annotation with Prokka version 1.14.5, AMR characterization with AMRFinderPlus version 3.11, and TB-specific analysis using TBProfiler version 6.2.0. MashTree version 1.2.1 was used for site-specific phylogenetic analysis of isolates, while Lyve-SET version 1.2.1 [24] was used for SNP analysis with *M. tuberculosis* H37Rv (NC_000962.3) set as the reference genome. Antimicrobial susceptibility test (AST) results for MTB isolates were provided by NSPHL and SFPHL.

## 5. Conclusions

In conclusion, the present study highlighted the suitability and robustness of a fully automated bacteria WGS assay for seamless, accurate detection and in-depth characterization of MTB clinical isolates and NTM reference isolates with high consistency. The assay demonstrated 100% concordance in species and sub-lineage classification and 92% (35 out of 38 isolates) concordance in genotypic vs. phenotypic AMR characterization. The WGS data generated will further propel research into the investigation of different MTB virulence factor mechanisms, such as secretion factors, cell surface components, enzymes involved in cellular metabolism, and transcriptional regulators [25]. This fully automated WGS solution, with a short turnaround time, can further aid in the surveillance and resolution of TB outbreaks in a timely manner. A significantly shorter (less than 2-day) turnaround time will increase actionability and patient safety by reducing and preventing MDR-MTB transmission and related fatalities, as well as reducing healthcare costs [26,27]. A fully automated bacteria WGS assay will be a valuable technology and tool to enhance the efficiency of diagnostic laboratories while supporting WHO’s vision for the elimination of this deadly disease. This study has implications for both government and nongovernmental organization resource allocation decisions and policy formulation, thereby establishing a favorable proposition for the adoption of a fully automated turnkey WGS solution for the decentralization of precision medicine and TB diagnostics.

## Figures and Tables

**Table 1:** Automated Bacterial WGS Assay Results for MTBs and NTMs.

Sample ID	Predicted Taxon	Sub-Lineage	Assembly Length (bp)	Number of Contigs	Estimated Depth of Coverage
NSPHL_Strain_8	*Mycobacterium tuberculosis*	1.2.1.2.1	4,353,471	127	119.63
NSPHL_Strain_9	*Mycobacterium tuberculosis*	1.2.1.2.1	4,372,144	122	60.37
NSPHL_Strain_10	*Mycobacterium tuberculosis*	4.1.2.1	4,316,704	142	33.78
NSPHL_Strain_11	*Mycobacterium tuberculosis*	4.1.1.1	4,344,545	118	100.73
NSPHL_Strain_12	*Mycobacterium tuberculosis*	4.1.1.3	4,335,708	124	58.77
NSPHL_Strain_13	*Mycobacterium tuberculosis*	1.2.1.2.1	4,356,191	139	62.57
NSPHL_Strain_14	*Mycobacterium tuberculosis*	3.1.2	4,343,589	125	114.74
NSPHL_Strain_15	*Mycobacterium tuberculosis*	4.8	4,335,730	115	52.41
NSPHL_Strain_16	*Mycobacterium tuberculosis*	1.2.1.2.1	4,326,334	239	20.66
NSPHL_Strain_17	*Mycobacterium tuberculosis*	4.1.2.1	4,326,395	159	34.55
36359772	*Mycobacterium tuberculosis*	2.2.1	4,288,293	189	45.65
36360342	*Mycobacterium tuberculosis*	1.1.1.1	4,312,674	222	33.05
36360344	*Mycobacterium tuberculosis*	2.2.1	4,257,211	278	33.06
36360347	*Mycobacterium tuberculosis*	4.6.2.2	4,258,952	267	32.12
36360353	*Mycobacterium tuberculosis*	4.1.2	4,322,326	233	34.83
36360355	*Mycobacterium tuberculosis*	4.1.2	4,203,142	593	21.84
36360361	*Mycobacterium tuberculosis*	1.1.1.1	4,306,182	247	34.21
36360364	*Mycobacterium tuberculosis*	1.1.1.1	4,314,878	195	45.81
36360369	*Mycobacterium tuberculosis*	4.1.2	4,292,556	272	36.49
36360376	*Mycobacterium tuberculosis*	4.1.2	4,300,124	293	31.64
36360377	*Mycobacterium tuberculosis*	1.2.1.2.1	4,360,550	150	100.91
36360388	*Mycobacterium tuberculosis*	4.6.2.2	4,314,959	121	98.35
Z008267	*Mycobacterium tuberculosis*	2.2.1	4,280,868	298	25.63
Z008268	*Mycobacterium tuberculosis*	2.2.1	4,322,819	159	43.21
Z008270	*Mycobacterium tuberculosis*	4.6.2.2	4,309,779	118	78.37
Z008271	*Mycobacterium tuberculosis*	1.2.1.2.1	4,355,604	136	58.3
Z008272	*Mycobacterium tuberculosis*	2.2.1	4,314,821	181	36.07
Z008273	*Mycobacterium tuberculosis*	1.2.1.2	4,341,999	153	50.4
Z008274	*Mycobacterium tuberculosis*	1.1.1.1	4,332,833	166	37.56
Z008275	*Mycobacterium tuberculosis*	2.2.1	4,319,340	196	35.28
Z008276	*Mycobacterium tuberculosis*	2.2.1.1	4,305,996	191	29.81
Z008277	*Mycobacterium tuberculosis*	1.1.1.1	4,348,679	120	92.59
Z008278	*Mycobacterium tuberculosis*	1.1.1.1	4,347,657	114	156.87
Z008279	*Mycobacterium tuberculosis*	4.6.2.2	4,321,629	139	38.43
ATCC_35734	*Mycobacterium tuberculosis*	La1.2.BCG	4,229,722	138	46.66
ATCC_35822D-2	*Mycobacterium tuberculosis*	4.9	4,285,606	122	60.58
NR-122	*Mycobacterium tuberculosis*	4.9	4,348,364	124	54.23
NR-59207	*Mycobacterium tuberculosis*	La1.8.1	4,242,260	183	24.48
NR-44263	*Mycobacteroides abscessus*	-	5,133,647	21	47.31
NR-44274	*Mycobacteroides abscessus*	-	5,188,746	30	37.27
NR-49658	*Mycobacterium canettii*	-	4,341,653	223	28.38
ATCC_6841	*Mycolicibacterium fortuitum*	-	6,279,033	47	43.01
ATCC_14470	*Mycobacterium gordonae*	-	7,368,456	278	27.74
NR-49070	*Mycobacterium palustre*	-	5,768,069	204	42.5

**Table 2:** Antimicrobial Resistance (AMR) Genotypic and Drug Class Profiles of MTB Isolates.

Sample ID	Sub-Lineage	Predicted AMR Drug Classes	Identified AMR Genes/Mutations	TB DR Type *
NSPHL_Strain_8	1.2.1.2.1	Aminoglycoside, Beta-Lactam, Lincosamide/Macrolide, Isoniazid, Ethionamide	aac(2′)-Ic, blaC, erm(37), fabG1_c.-15C>T	HR-TB
NSPHL_Strain_9	1.2.1.2.1	Aminoglycoside, Beta-Lactam, Lincosamide/Macrolide, Isoniazid	aac(2′)-Ic, blaC, erm(37), katG_p.Ser315Thr	HR-TB
NSPHL_Strain_10	4.1.2.1	Aminoglycoside, Beta-Lactam, Lincosamide/Macrolide	aac(2′)-Ic, blaC, erm(37)	Sensitive
NSPHL_Strain_11	4.1.1.1	Aminoglycoside, Beta-Lactam, Lincosamide/Macrolide	aac(2′)-Ic, blaC, erm(37)	Sensitive
NSPHL_Strain_12	4.1.1.3	Aminoglycoside, Beta-Lactam, Lincosamide/Macrolide, Isoniazid, Fluoroquinolones, Ethionamide	aac(2′)-Ic, blaC, erm(37), fabG1_c.-15C>T, gyrA_p.Ala90Val	HR-TB
NSPHL_Strain_13	1.2.1.2.1	Aminoglycoside, Beta-Lactam, Lincosamide/Macrolide, Rifampicin	aac(2′)-Ic, blaC, erm(37), rpoB_p.Asp435Tyr	RR-TB
NSPHL_Strain_14	3.1.2	Aminoglycoside, Beta-Lactam, Lincosamide/Macrolide, Isoniazid, Ethambutol, Streptomycin	aac(2′)-Ic, blaC, erm(37), embB_p.Met306Val, gid_c.115delC, katG_p.Ser315Thr	HR-TB
NSPHL_Strain_15	4.8	Aminoglycoside, Beta-Lactam, Lincosamide/Macrolide, Isoniazid, Ethambutol	aac(2′)-Ic, blaC, erm(37), inhA_c.-154G>A	HR-TB
NSPHL_Strain_16	1.2.1.2.1	Aminoglycoside, Beta-Lactam, Lincosamide/Macrolide, Streptomycin	aac(2′)-Ic, blaC, erm(37), gid_c.115delC	Other
NSPHL_Strain_17	4.1.2.1	Aminoglycoside, Beta-Lactam, Lincosamide/Macrolide, Rifampicin	aac(2′)-Ic, blaC, erm(37), rpoB_p.Ser450Leu	RR-TB
36359772	2.2.1	Aminoglycoside, Beta-Lactam, Lincosamide/Macrolide	aac(2′)-Ic, blaC, erm(37)	Sensitive
36360342	1.1.1.1	Aminoglycoside, Beta-Lactam	aac(2′)-Ic, blaC	Sensitive
36360344	2.2.1	Aminoglycoside, Beta-Lactam, Lincosamide/Macrolide	aac(2′)-Ic, blaC, erm(37)	Sensitive
36360347	4.6.2.2	Aminoglycoside, Beta-Lactam, Lincosamide/Macrolide	aac(2′)-Ic, blaC, erm(37)	Sensitive
36360353	4.1.2	Aminoglycoside, Beta-Lactam, Lincosamide/Macrolide	aac(2′)-Ic, blaC, erm(37)	Sensitive
36360355	4.1.2	Aminoglycoside, Beta-Lactam, Lincosamide/Macrolide, Pyrazinamide	aac(2′)-Ic, blaC, erm(37), pncA_p.Gly132Asp	Other
36360361	1.1.1.1	Aminoglycoside, Beta-Lactam	aac(2′)-Ic, blaC	Sensitive
36360364	1.1.1.1	Aminoglycoside, Beta-Lactam	aac(2′)-Ic, blaC	Sensitive
36360369	4.1.2	Aminoglycoside, Beta-Lactam, Lincosamide/Macrolide	aac(2′)-Ic, blaC, erm(37)	Sensitive
36360376	4.1.2	Aminoglycoside, Beta-Lactam, Lincosamide/Macrolide	aac(2′)-Ic, blaC, erm(37)	Sensitive
36360377	1.2.1.2.1	Aminoglycoside, Beta-Lactam, Lincosamide/Macrolide, Isoniazid	aac(2′)-Ic, blaC, erm(37), fabG1_c.-15C>T	HR-TB
36360388	4.6.2.2	Aminoglycoside, Beta-Lactam, Lincosamide/Macrolide	aac(2′)-Ic, blaC, erm(37)	Sensitive
Z008267	2.2.1	Aminoglycoside, Beta-Lactam, Lincosamide/Macrolide	aac(2′)-Ic, blaC, erm(37)	Sensitive
Z008268	2.2.1	Aminoglycoside, Beta-Lactam, Lincosamide/Macrolide	aac(2′)-Ic, blaC, erm(37)	Sensitive
Z008270	4.6.2.2	Aminoglycoside, Beta-Lactam, Lincosamide/Macrolide	aac(2′)-Ic, blaC, erm(37)	Sensitive
Z008271	1.2.1.2.1	Aminoglycoside, Beta-Lactam, Lincosamide/Macrolide	aac(2′)-Ic, blaC, erm(37)	Sensitive
Z008272	2.2.1	Aminoglycoside, Beta-Lactam, Lincosamide/Macrolide, Pyrazinamide	aac(2′)-Ic, blaC, erm(37), kasA_p.Gly387Asp	HR-TB
Z008273	1.2.1.2	Aminoglycoside, Beta-Lactam, Lincosamide/Macrolide	aac(2′)-Ic, blaC, erm(37)	Sensitive
Z008274	1.1.1.1	Aminoglycoside, Beta-Lactam	aac(2′)-Ic, blaC	Sensitive
Z008275	2.2.1	Aminoglycoside, Beta-Lactam, Lincosamide/Macrolide	aac(2′)-Ic, blaC, erm(37)	Sensitive
Z008276	2.2.1.1	Aminoglycoside, Beta-Lactam, Lincosamide/Macrolide	aac(2′)-Ic, blaC, erm(37)	Sensitive
Z008277	1.1.1.1	Aminoglycoside, Beta-Lactam	aac(2′)-Ic, blaC	Sensitive
Z008278	1.1.1.1	Aminoglycoside, Beta-Lactam	aac(2′)-Ic, blaC	Sensitive
Z008279	4.6.2.2	Aminoglycoside, Beta-Lactam, Lincosamide/Macrolide	aac(2′)-Ic, blaC, erm(37)	Sensitive
ATCC_35734	La1.2.BCG	Aminoglycoside, Beta-Lactam, Lincosamide/Macrolide, Pyrazinamide	aac(2′)-Ic, blaC, erm(37), pncA_p.His57Asp	Other
ATCC_35822D-2	4.9	Aminoglycoside, Beta-Lactam, Lincosamide/Macrolide, Isoniazid	aac(2′)-Ic, blaC, erm(37), ahpC_c.-39C>T, ahpC_c.-54C>T, katG_c.-11139_*36437del	HR-TB
NR-122	4.9	Aminoglycoside, Beta-Lactam, Lincosamide/Macrolide	aac(2′)-Ic, blaC, erm(37)	Sensitive
NR-59207	La1.8.1	Aminoglycoside, Beta-Lactam, Lincosamide/Macrolide, Pyrazinamide	aac(2′)-Ic, blaC, erm(37), pncA_p.His57Asp	Other

* DR: Drug Resistance; HR-TB: Isoniazid-Resistant TB; RR-TB: Rifampicin-Resistant TB.

**Table 3:** Reproducibility Study Validated the Robustness of the Fully Automated Bacteria WGS Assay.

Sample ID	Sample Size	Assembly Length (bp) Mean ± S.D.	Number of Contigs Mean ± S.D.	Mean Q Scores Mean ± S.D.	Mean Read Length (bp) Mean ± S.D.
NSPHL_Strain_8 (MTB)	4	4,335,605 ± 2228	172.67 ± 8.74	36.12 ± 0.06	147.43 ± 0.78
NSPHL_Strain_12 (MTB)	3	4,305,569 ± 5272	201.00 ± 8.28	36.19 ± 0.01	147.46 ± 0.34
NSPHL_Strain_17 (MTB)	3	4,324,000 ± 5569	166.67 ± 2.42	36.08 ± 0.04	147.49 ± 0.81
ATCC_35734 (MTB)	3	4,224,952 ± 4199	141.67 ± 5.51	35.56 ± 0.05	145.82 ± 0.95
ATCC_35822D-2 (MTB)	5	4,282,387 ± 3431	129.00 ± 4.95	35.49 ± 0.05	144.77 ± 1.93
NR-122 (MTB)	16	4,336,706 ± 7704	136.47 ± 10.11	35.49 ± 0.34	147.21 ± 1.33
NR-59207 (MTB)	3	4,238,013 ± 6183	192.67 ± 8.50	34.71 ± 0.00	145.99 ± 0.57
ATCC_6841 (NTM)	3	6,274,061 ± 4522	56.67 ± 9.07	35.73 ± 0.09	146.60 ± 1.18
NR-44263 (NTM)	4	5,130,504 ± 3015	29.25 ± 8.88	35.73 ± 0.08	144.11 ± 1.68
NR-44274 (NTM)	4	5,189,594 ± 3121	31.25 ± 7.09	35.84 ± 0.09	147.98 ± 0.11
NR-49070 (NTM)	2	5,768,681 ± 865	205.00 ± 1.41	36.03 ± 0.61	144.26 ± 3.20

**Table 4:** Concordance Between Genotypic and Phenotypic AMR Profiles of MTB Isolates from NSPHL.

Sample ID	Sub-Lineage	Predicted AMR Drug Classes	Antimicrobial Susceptibility Test (AST)				
			Ethambutol 5.0 μg/mL	Isoniazid 0.1 μg/mL	Isoniazid 0.4 μg/mL	Rifampin 1.0 μg/mL	Pyrazinamide 100 μg/mL
NSPHL_Strain_8	1.2.1.2.1	Aminoglycoside, Beta-Lactam, Lincosamide/Macrolide, Isoniazid, Ethionamide	Sensitive	Resistant	Resistant	Sensitive	Sensitive
NSPHL_Strain_9	1.2.1.2.1	Aminoglycoside, Beta-Lactam, Lincosamide/Macrolide, Isoniazid	Sensitive	Resistant	Resistant	Sensitive	Sensitive
NSPHL_Strain_10	4.1.2.1	Aminoglycoside, Beta-Lactam, Lincosamide/Macrolide	Not Available				
NSPHL_Strain_11	4.1.1.1	Aminoglycoside, Beta-Lactam, Lincosamide/Macrolide	Sensitive	Sensitive	Sensitive	Sensitive	Sensitive
NSPHL_Strain_12	4.1.1.3	Aminoglycoside, Beta-Lactam, Lincosamide/Macrolide, Isoniazid, Fluoroquinolones, Ethionamide	Sensitive	Resistant	Sensitive	Sensitive	Sensitive
NSPHL_Strain_13	1.2.1.2.1	Aminoglycoside, Beta-Lactam, Lincosamide/Macrolide, Rifampicin	Sensitive	Sensitive	Sensitive	Resistant	Sensitive
NSPHL_Strain_14	3.1.2	Aminoglycoside, Beta-Lactam, Lincosamide/Macrolide, Isoniazid, Ethambutol, Streptomycin	Sensitive	Resistant	Resistant	Sensitive	Sensitive
NSPHL_Strain_15	4.8	Aminoglycoside, Beta-Lactam, Lincosamide/Macrolide, Isoniazid, Ethambutol	Sensitive	Resistant	Sensitive	Sensitive	Sensitive
NSPHL_Strain_16	1.2.1.2.1	Aminoglycoside, Beta-Lactam, Lincosamide/Macrolide, Streptomycin	Not Available				
NSPHL_Strain_17	4.1.2.1	Aminoglycoside, Beta-Lactam, Lincosamide/Macrolide, Rifampicin	Not Available				

**Table 5:** Bacterial Strains Used in This Study.

Organism	Sample ID	Source
***Mycobacterium tuberculosis* strains**		
*Mycobacterium tuberculosis* ^3^	NSPHL Strain 8	NSPHL
*Mycobacterium tuberculosis* ^3^	NSPHL Strain 9	NSPHL
*Mycobacterium tuberculosis* ^3^	NSPHL Strain 10	NSPHL
*Mycobacterium tuberculosis* ^3^	NSPHL Strain 11	NSPHL
*Mycobacterium tuberculosis* ^3^	NSPHL Strain 12	NSPHL
*Mycobacterium tuberculosis* ^3^	NSPHL Strain 13	NSPHL
*Mycobacterium tuberculosis* ^3^	NSPHL Strain 14	NSPHL
*Mycobacterium tuberculosis* ^3^	NSPHL Strain 15	NSPHL
*Mycobacterium tuberculosis* ^3^	NSPHL Strain 16	NSPHL
*Mycobacterium tuberculosis* ^3^	NSPHL Strain 17	NSPHL
*Mycobacterium tuberculosis* ^4^	36359772	SFPHL
*Mycobacterium tuberculosis* ^4^	36360342	SFPHL
*Mycobacterium tuberculosis* ^4^	36360344	SFPHL
*Mycobacterium tuberculosis* ^4^	36360347	SFPHL
*Mycobacterium tuberculosis* ^4^	36360353	SFPHL
*Mycobacterium tuberculosis* ^4^	36360355	SFPHL
*Mycobacterium tuberculosis* ^4^	36360361	SFPHL
*Mycobacterium tuberculosis* ^4^	36360364	SFPHL
*Mycobacterium tuberculosis* ^4^	36360369	SFPHL
*Mycobacterium tuberculosis* ^4^	36360376	SFPHL
*Mycobacterium tuberculosis* ^4^	36360377	SFPHL
*Mycobacterium tuberculosis* ^4^	36360388	SFPHL
*Mycobacterium tuberculosis* ^5^	Z008267	SFPHL
*Mycobacterium tuberculosis* ^5^	Z008268	SFPHL
*Mycobacterium tuberculosis* ^5^	Z008270	SFPHL
*Mycobacterium tuberculosis* ^5^	Z008271	SFPHL
*Mycobacterium tuberculosis* ^5^	Z008272	SFPHL
*Mycobacterium tuberculosis* ^5^	Z008273	SFPHL
*Mycobacterium tuberculosis* ^5^	Z008274	SFPHL
*Mycobacterium tuberculosis* ^5^	Z008275	SFPHL
*Mycobacterium tuberculosis* ^5^	Z008276	SFPHL
*Mycobacterium tuberculosis* ^5^	Z008277	SFPHL
*Mycobacterium tuberculosis* ^5^	Z008278	SFPHL
*Mycobacterium tuberculosis* ^5^	Z008279	SFPHL
*Mycobacterium tuberculosis*, H37Ra ^1^	NR-122	BEI Resources
*Mycobacterium tuberculosis*, Strain TMC 303 ^2^	ATCC_35822D-2	ATCC
*Mycobacterium tuberculosis* variant *bovis* BCG ^1^	ATCC_35734	ATCC
*Mycobacterium bovis*, Strain 95-1315 ^2^	NR-59207	BEI Resources
**Non-tuberculosis *Mycobacterium* strains**		
*Mycobacteroides abscessus*, 4530 ^1^	NR-44274	BEI Resources
*Mycobacteroides abscessus bolletii*, MA 1948 ^1^	NR-44263	BEI Resources
*Mycobacterium canettii*, Strain NLA000017120 ^2^	NR-49658	BEI Resources
*Mycobacterium gordonae* ^1^	ATCC_14470	ATCC
*Mycobacterium palustre*, FI-05088 ^1^	NR-49070	BEI Resources
*Mycolicibacterium fortuitum*, Strain TMC 1529 ^1^	ATCC_6841	ATCC

^1^ DNA was extracted by Clear Labs, Inc. using a proprietary, in-house-developed extraction protocol. ^2^ Genomic DNA was obtained from the culture collections (ATCC or BEI Resources). ^3^ DNA was extracted by the Nevada State Public Health Laboratory (NSPHL) using the Promega Maxwell RSC Cultured Cell Kit. ^4^ DNA was extracted by the San Francisco Public Health Laboratory (SFPHL) using the Roche MagNA Pure 24 Total NA Isolation Kit. ^5^ DNA was extracted by San Francisco Public Health Laboratory (SFPHL) using the QIAGEN EZ1&2 Virus Mini Kit.

## Data Availability

Sequence data obtained from the 34 clinical *Mycobacterium tuberculosis* isolates in this study are publicly available under BioProject PRJNA1129189. The Whole Genome Shotgun project has been deposited at DDBJ/ENA/GenBank under the accession numbers detailed in Table S4.

## Supplementary Materials

The following supporting information can be downloaded at: https://www.mdpi.com/article/10.3390/ijms25147909/s1.
